# Exploiting Fungal Virulence-Regulating Transcription Factors As Novel Antifungal Drug Targets

**DOI:** 10.1371/journal.ppat.1004936

**Published:** 2015-07-16

**Authors:** Yong-Sun Bahn

**Affiliations:** Department of Biotechnology, College of Life Science and Biotechnology, Yonsei University, Seoul, Republic of Korea; Geisel School of Medicine at Dartmouth, UNITED STATES

## Are Current Antifungal Drug Targets Sufficient for the Treatment of Systemic Mycosis?

Systemic and invasive mycoses caused by primary and opportunistic fungal pathogens have been emerging as global problems because of the increase in the number of immunocompromised individuals, due to solid-organ transplants, anti-cancer chemotherapy, and extended human lifespan. A recent report estimated that fungal pathogens, such as *Cryptococcus neoformans*, *Candida albicans*, and *Aspergillus fumigatus*, are responsible for more than 1 million deaths annually [1]. Despite this, the availability of antifungal drugs or targets for antifungal drug development are very limited. This is unlike the situation of bacterial pathogens and, to an extent, the eukaryotic parasites (which is serious enough) because the animals share a more recent common ancestor with the fungi than other pathogens. Ergosterol and its biosynthetic enzymes are the most popular antifungal drug targets because of the structural distinguishability of ergosterol from cholesterol in mammalian cell membranes. Polyene macrolides directly bind to ergosterol and generate lethal transmembrane channels that leak essential cellular ions and perturb osmotic balances, which leads to cell death [2]. Azole and allylamine derivatives are inhibitors of the ergosterol biosynthetic pathway that inhibit 14α-demethylase and squalene epoxidase, respectively, eventually leading to the accumulation of toxic precursors of ergosterol in the cell membrane and subsequent impairment of membrane integrity [3]. Another promising antifungal drug target is the fungal cell wall. Echinocandin inhibits β-1,3-glucan synthase and impairs cell wall integrity [4]. Nucleotide biosynthesis is also, somewhat unexpectedly, an appropriate antifungal drug target. For example, flucytosine itself does not have antifungal activity; however, after its uptake into cells, it is rapidly converted to 5-fluorouracil, which inhibits DNA and protein synthesis by cytosine deaminase, absent in humans [5]. However, all these antifungal drugs have problems, such as toxicity (e.g., hepatotoxicity and nephrotoxicity), frequent emergence of resistance, and a limited spectrum [2–5]. To overcome these problems, novel antifungal drug targets and drugs need to be discovered and developed.

## Can Transcription Factors Serve As Novel Antifungal Drug Targets?

**Fig 1:**
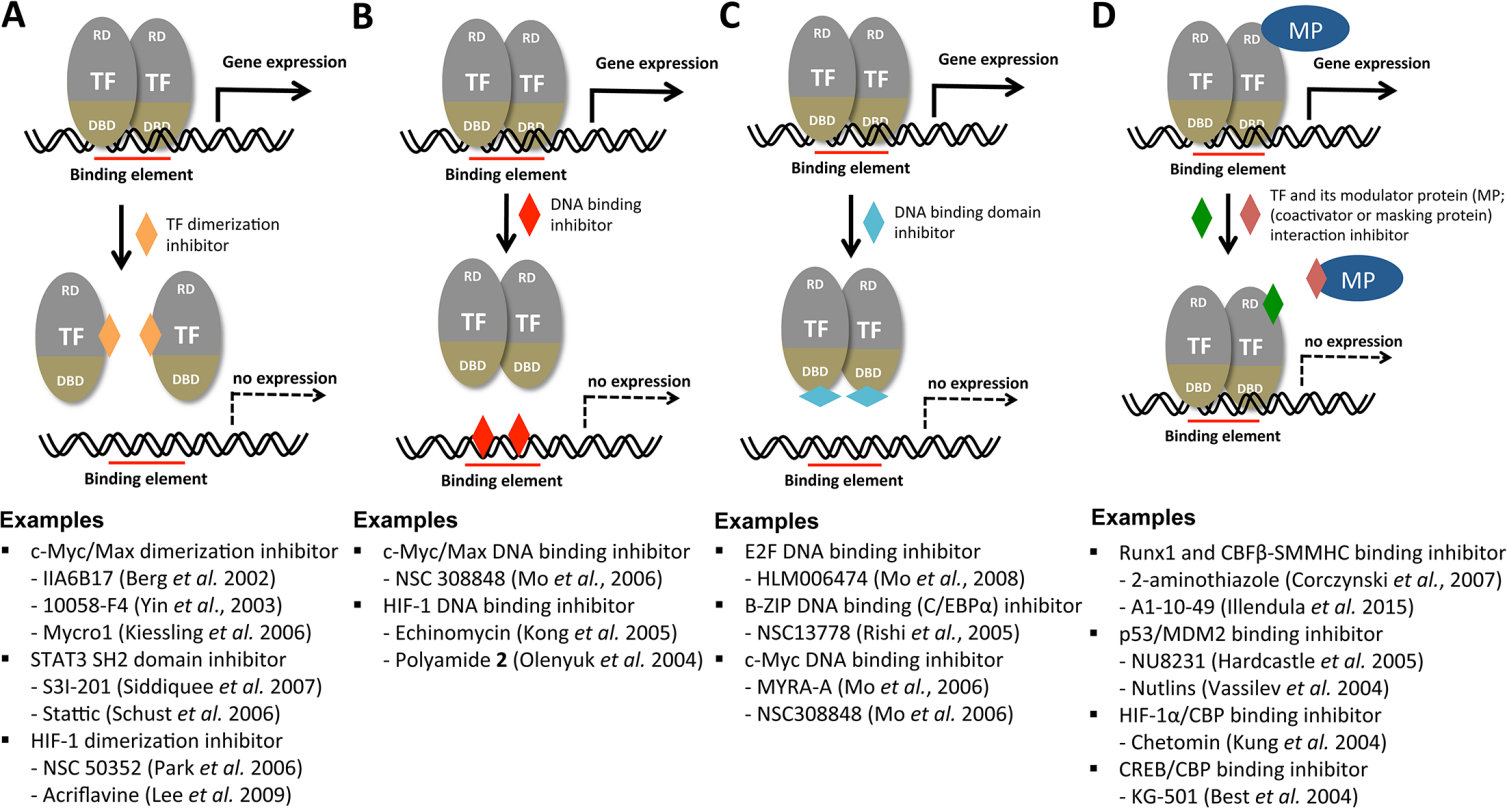
Potential mechanisms for the chemical modulation of transcription factors. Transcription factors (TFs) are illustrated as a modular structure consisting of a DNA-binding domain (DBD) and regulatory domain (RD). MP indicates any modular protein (coactivator or masking protein). Although TFs are assumed to be homodimers here, they could have monomeric, multimeric, heterodimeric, or other structural configurations. In this overview, chromatin-remodeling enzymes, RNA polymerase and its multiple cofactors, all of which are required for general transcriptional induction of a gene, are not illustrated. The function of a TF can be perturbed by inhibiting TF dimerization (A), masking the DNA binding element (B), TF DNA binding domain (C), or inhibiting interaction between the TF and its modulator protein (D). Detailed reference information for each developing or developed TF inhibitor under each category can be obtained from the following review articles: [7–9].

Transcription factors (TFs) are attractive as novel antifungal drug targets because they are evolutionarily divergent between fungi and humans (even among fungal species) and hence can be exploited as selective drug targets. In general, TFs have been considered poor drug targets because drugs that target them would have to specifically disrupt protein–nucleic acid or protein–protein interactions, rather than simply binding to an active pocket in an enzyme [6]. However, accumulated evidence thus far strongly demonstrates that TFs are chemically tractable [7–9]. Natural or synthetic chemicals or peptidomimetics have been identified based on their ability to inhibit hetero- or homo-dimerization of TFs, TF-binding DNA elements, DNA-binding domains of TFs, or the interaction between a TF and its essential modulating proteins, as summarized in Fig 1. Among the TF-targeting small molecules listed in Fig 1, nutlins, which are specific MDM2-p53 antagonists, are being evaluated in the early clinical trials for cancer treatment [10]. To this end, chemical biologists employ yeast two-hybrid or one-hybrid reporter assays; fluorescence resonance energy transfer (FRET) assays; small-molecule microarrays; or structure-based, computational, virtual, drug-protein docking simulations as screening tools [7–9]. Besides the approach using small molecules, other approaches using polyamides, small interfering RNAs, TF decoy oligonucleotides, and synthetic peptides have been employed to inhibit TFs [7–9]. Therefore, along with these recent technical breakthroughs in chemical biology fields, structural information and elucidation of the DNA-binding element and any co-regulators for a given virulence-regulating TF could pave the way for developing novel antifungal agents and therapeutic methods.

## How Much Have Transcription Factors Been Functionally Characterized in Human Fungal Pathogens?

Due to the reasons described above, the functional characterization of fungal TFs, particularly their role in pathogenicity, has been in high demand. To this end, several large-scale functional genomic analyses of fungal TFs have been independently performed in two major human fungal pathogens, *C*. *albicans* and *C*. *neoformans*. Nobile and Mitchell generated 83 TF mutants of *C*. *albicans* and addressed their roles in biofilm formation [11]. Homann et al. characterized the in vitro functions of 166 TFs under 50 different growth conditions [12]. However, neither study directly addressed their roles in the pathogenicity of *C*. *albicans*. Subsequently, Noble et al. generated homozygous mutant strains for 674 genes and discovered 115 infectivity-attenuated mutants [13]. Among these, 13 virulence-regulating TFs were discovered. In *C*. *neoformans*, three large-scale functional analyses of TFs have been performed thus far. Liu et al. generated gene-deletion mutants for 58 TFs as a part of large-scale gene deletion project and discovered 20 TFs that are involved in the lung infectivity of *C*. *neoformans* [14]. More recently, Jung et al. generated gene-deletion mutants for 155 non-essential, sequence-specific DNA-binding TFs and evaluated their virulence and infectivity potentials in both insect and murine models [15]. They discovered that 45 TFs are involved in either virulence or infectivity of *C*. *neoformans*. Furthermore, for the purpose of constructing model-driven comprehensive transcriptional networks for capsule biosynthesis in *C*. *neoformans*, Maier et al. analyzed 41 TF mutants with altered capsule production and found 10 infectivity-related TFs [16]. Besides these large-scale studies, a number of studies characterizing the function of individual TFs in the pathogenicity of *C*. *albicans* and *C*. *neoformans* have been performed. Therefore, a plethora of information is available for selecting potential TF targets that could be exploited for the development of TF-targeting antifungal drugs.

## What Transcription Factors Could Be Broad-Spectrum Antifungal Drug Targets?

**Fig 2:**
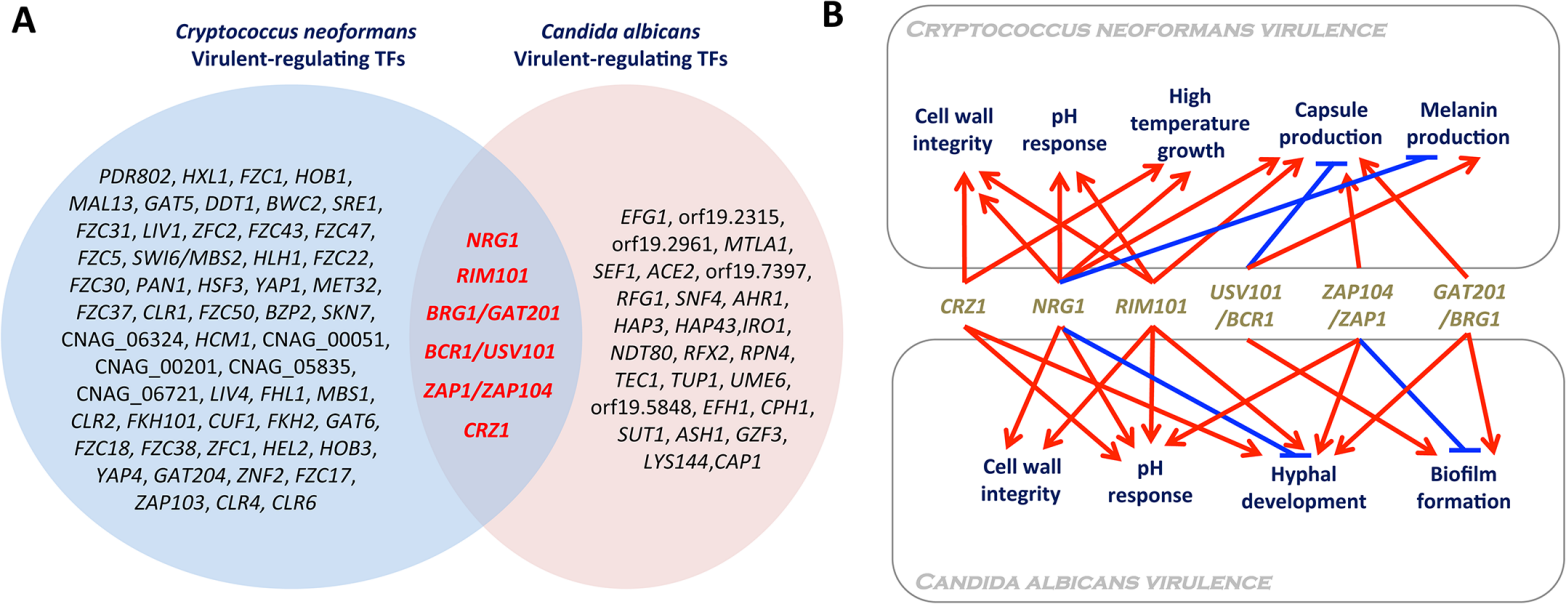
Virulence-regulating transcription factors in *Candida albicans* and *Cryptococcus neoformans*. (A) The Venn diagram demonstrates TF genes whose deletion affects (either reduces or enhances) the pathogenicity (infectivity and/or virulence) of *C*. *albicans* and *C*. *neoformans*. List of virulence-regulating TFs was mainly retrieved from large-scale functional genetic studies of *C*. *neoformans* and *C*. *albicans* TFs [11,13–16] and a curated *C*. *albicans* genome database (http://www.candidagenome.org/). (B) Virulence-related cellular functions of TFs that could be exploited as broad-spectrum antifungal drug targets. Deletion of *NRG1*, *BRG1/GAT201*, *BCR1/USV101*, *ZAP1/ZAP101*, or *CRZ1* is known to reduce the virulence of *C*. *albicans* and *C*. *neoformans*. Deletion of *RIM101* enhances the virulence of *C*. *neoformans* but reduces the virulence of *C*. *albicans*.

Comparison of functional TF analysis data of both *C*. *albicans* and *C*. *neoformans* provides an insight into what kinds of TFs could be exploited as broad- or narrow-spectrum antifungal drug targets. TFs that have been demonstrated to be involved in infectivity or virulence of *C*. *albicans* and *C*. *neoformans* are summarized in Fig 2. The following six TFs were found to be commonly involved in the virulence of both fungal pathogens: Crz1, Nrg1, Rim101, Bcr1/Usv101, Zap1/Zap104, and Brg1/Gat201.

Interestingly, except Brg1/Gat201, Crz1, Nrg1, Rim101, Bcr1/Usv101, and Zap1/Zap104, all encode C_2_H_2_ zinc finger-type TFs. Crz1 is a direct downstream target of fungal calcineurin, a Ca^2+^/calmodulin-dependent protein phosphatase that modulates ion homeostasis, pH response, cell wall integrity, thermotolerance, developmental processes, and/or the virulence of a variety of human fungal pathogens including *C*. *albicans*, *C*. *neoformans*, and *A*. *fumigatus* [17]. The role of Rim101 and Nrg1 is similar to that of Crz1 in cell wall integrity; deletion of *RIM101* and *NRG1* alters cell wall integrity in both *C*. *neoformans* [18,19] and *C*. *albicans* [20,21]. Furthermore, Rim101 and Nrg1 are functionally well connected for modulating cellular pH responses in human fungal pathogens. Notably, however, deletion of *RIM101* reduces the virulence of *C*. *albicans* [22] but increases the virulence of *C*. *neoformans* due to abnormal stimulation of immune responses [18,23], suggesting that Rim101 may not be a good broad-spectrum antifungal drug target. Rim101 negatively regulates Nrg1, which represses the expression of *ENA1*, a Na^+^/K^+^ transporter, in both *C*. *albicans* and *C*. *neoformans* [24,25]. In *C*. *neoformans* particularly, deletion of *ENA1* completely abolishes virulence [24]. Therefore, their roles in pH response and cation homeostasis may also affect the virulence of fungal pathogens.

The function of Usv101 has been recently characterized in *C*. *neoformans* by Jung et al. [15] and Maier et al. [16]. The *usv101*∆ mutant had a reduced pulmonary infectivity, whereas it was shown to have an increased lung infectivity by Liu et al. [14]. This discrepancy seems to result from differences in the parental strains used for each study. The *usv101*∆ mutant exhibits increased capsule production, but decreased melanin synthesis, in the H99S and KN99α backgrounds. The latter phenotypic trait may be responsible for its reduced infectivity. By contrast, the CMO18 strain is inherently defective in melanin synthesis. Therefore, only capsule enhancement could be reflected in the increased lung infectivity of the *usv101*∆ mutant in the CMO18 strain. The closest *C*. *albicans* ortholog of Usv101 is Bcr1, which regulates biofilm formation and expression of cell-surface adherence genes [11]. Deletion of *ZAP104* severely diminishes both capsule production and mating efficiency in *C*. *neoformans* [15], among which the former trait may affect the virulence of *C*. *neoformans*. The closest *C*. *albicans* ortholog of Zap104 is Zap1/Csr1. Zap1 is not only a regulator of zinc homeostasis but also regulates filamentous growth and biofilm maturation of *C*. *albicans* in positive and negative manners, respectively [26].

*BRG1/GAT201* encodes a GATA-type TF. In *C*. *neoformans*, Gat201 regulates both capsule-dependent and-independent antiphagocytic mechanisms in *C*. *neoformans* [27]. Deletion of *GAT201* severely reduces the lung infectivity of *C*. *neoformans* [14,15]. Surprisingly, Gat201 regulates about 16% of the *C*. *neoformans* genome, suggesting that it is one of the master regulators [27]. The closest *C*. *albicans* ortholog of Gat201 is Brg1 (also known as Gat2), which plays a major role in hyphal elongation in *C*. *albicans* by recruiting the histone deacetylase Hda1 to the promoters of hypha-specific genes, while the cAMP/PKA-dependent removal of Nrg1 is required for hyphal initiation [28]. Furthermore, Brg1 also promotes biofilm development [29]. The fact that both overexpression and deletion of *BRG1* attenuated the virulence of *C*. *albicans* [30,31] suggests that orchestrated in vivo regulation of *BRG1* is critical for its pathogenicity.

Notably, mutants of Crz1, Nrg1, Usv101, Gat201, and Zap104 exhibit increased susceptibility to polyene or azole drugs [15], suggesting that these broad-spectrum target TFs could also be exploited as combination therapeutic targets of antifungal drugs that are already clinically available. However, regardless of orthologous relationship for the common virulence-regulating TFs between *C*. *neoformans* and *C*. *albicans*, the homologous region is mainly restricted to the DNA binding region. Therefore, it will be still challenging to develop a broad-spectrum antifungal agent that specifically targets the TFs.

## How About Exploiting Narrow-Spectrum Antifungal Drug Targets?

Although broad-spectrum antifungal drugs are generally favored from an industrial perspective, they have some potential drawbacks. For example, any known or unknown commensal or mutualistic fungal species in humans could be broadly affected, which may affect the balance of the normal microflora and cause secondary infection with other unwanted pathogens. In fact, *C*. *albicans* is part of the normal microflora in the gastrointestinal tract of healthy individuals. Therefore, if the identity of a fungal pathogen could be determined in the early stage of mycoses, pathogen-specific, narrow-spectrum targets could be even more optimal, and any drugs targeting such TFs would be expected to have less toxic effects (Fig 2). Several evolutionarily divergent, virulence-regulating TFs could be exploited as narrow-spectrum antifungal drugs.

Among many narrow-spectrum TF target candidates, Efg1 is the best characterized in *C*. *albicans*. Efg1, which is mainly controlled by the cAMP/PKA pathway, controls hyphal development and white-opaque phenotypic switching, both of which are critically involved in the pathogenicity of *C*. *albicans* [32]. In *C*. *neoformans*, *HXL1*, which is a bona fide downstream transcription factor of the Ire1 kinase in the unfolded-protein response pathway, is most notable, as its structure is evolutionarily divergent from its counterpart in humans (Xbp1) and its deletion completely abolishes the virulence of *C*. *neoformans* [33]. Another major benefit of targeting Efg1 or Hxl1 is that their inhibition strongly enhances the susceptibility to azole drugs [33,34], suggesting that they could be exploited as both single and combination therapeutic methods.

## Perspectives

As large-scale functional genomics data of fungal TFs become more readily available and their roles in fungal pathogenicity are uncovered, chemical biologists will become more interested in developing TF-targeting antifungal agents. To provide an efficient drug screening system exploiting virulence-regulating TFs, the following data need to be accumulated: (1) identification of direct DNA-binding elements; (2) structural characterization and functional domain analysis; (3) identification of any essential interacting partners, particularly co-activators; and (4) identification of any upstream regulators and downstream effector proteins. The core consensus DNA-binding motif for Crz1, CDRE (calcineurin-dependent response element), has been shown to be present in some fungi [17]. Recently, Maier et al. identified DNA-binding motifs for Gat201, Nrg1, and Usv101 by chromatin immunoprecipitation-sequence analysis in *C*. *neoformans* [16]. This information will be useful in constructing any drug-screening system for identifying direct inhibitors of TF-DNA interactions. In conclusion, a further in-depth molecular and genetic analysis of the virulence-regulating TFs will provide new insight into developing novel classes of antifungal agents that could resolve the problems associated with the currently available drugs.
